# Loosely synchronized activation of anterior cingulate cortical neurons for scratching response during histamine-induced itch

**DOI:** 10.1186/s13041-023-01037-7

**Published:** 2023-06-13

**Authors:** Chiwoo Lee, Jihae Oh, Jae-Hyung Lee, Bong-Kiun Kaang, Hyoung-Gon Ko

**Affiliations:** 1 Department of Biological Sciences, College of Natural Sciences, Seoul National University, 1 Gwanak-ro, Gwanak-gu, Seoul, 08826 South Korea; 2 Department of Oral Microbiology, College of Dentistry, Kyung Hee University, Seoul, 02447 South Korea; 3 Department of Anatomy and Neurobiology, School of Dentistry, Kyungpook National University, 2177 Dalgubeol-daero, Daegu, 41940 South Korea

**Keywords:** Anterior cingulate cortex, Itch, In vivo calcium imaging

## Abstract

Itch is a distinctive sensation that causes a specific affection and scratching reaction. The anterior cingulate cortex (ACC) has been linked to itch sensation in numerous studies; however, its precise function in processing pruritic inputs remains unknown. Distinguishing the precise role of the ACC in itch sensation can be challenging because of its capacity to conduct heterologous neurophysiological activities. Here, we used in vivo calcium imaging to examine how ACC neurons in free-moving mice react to pruritogenic histamine. In particular, we focused on how the activity of the ACC neurons varied before and after the scratching response. We discovered that although the change in neuronal activity was not synchronized with the scratching reaction, the overall activity of itch-responsive neurons promptly decreased after the scratching response. These findings suggest that the ACC does not directly elicit the feeling of itchiness.

**Supplementary Information:**

The online version contains supplementary material available at 10.1186/s13041-023-01037-7.

Itchy sensations serve to alert higher living organisms to external stimuli with potentially life-threatening factors, along with pain. In the periphery, itch-specific primary sensory neurons are activated by pruritic stimuli, which sequentially activate interneurons in the spinal cord or trigeminal nucleus. There is growing evidence that certain neuronal populations transmit pruritic stimuli to the spinal cord. However, which brain circuit is responsible for itch perception remains unknown [1]. The brain region or pathway through which pruriceptive stimuli are transmitted is largely similar to that through which pain is transmitted; however, recent studies have shown the presence of specific neurons in the brain that are implicated in the processing of pruriceptive stimuli [2, 3]. In the amygdala, specific neuronal populations are activated by various pruritogens that mediate pruritogen-induced scratching and anxiety [4]. In addition, GABAergic neurons in the ventral tegmental area (VTA) are activated within a certain time window of itching perception and enhance histamine-induced scratching behavior in mice [5]. These itch-specific neurons are also found in the ventrolateral orbital cortex (VLO) and play a role in itch-induced scratching behavior but not in nociception-induced defensive behavior [6]. These studies suggest that in the brain, the inherent neuronal population and circuit underlie itch perception, apart from other sensations, including pain.

The anterior cingulate cortex (ACC) is a subregion of the prefrontal cortex. One of the features of the ACC is its implication in a wide range of functions, including emotion, sensory information processing, and remote memory recall [7–10]. Regarding sensory information processing, it is well known that the ACC is specifically involved in the affective component of sensation. Previous studies have shown that the ACC is activated in response to nociceptive or pruriceptive stimuli in rodents and humans [3, 11–13]. Inhibition of excitatory neurons in the ACC reduces scratching responses against chronic itching conditions, while inhibition of inhibitory neurons has the opposite effect [14]. These studies have shown the involvement of the ACC in the processing of pruriceptive stimuli; however, the mechanism by which the ACC processes pruriceptive stimuli remains largely unknown. Thus, in this study, we investigated the dynamic activation patterns of ACC neurons in response to pruritogen and histamine using in vivo calcium imaging in free-moving mice.

To monitor the dynamic changes in ACC neuronal activity during itching perception, we used a 1p real-time calcium imaging system in a total of 5 mice. AAV2/1-EF1α-GCaMP6f were injected into the ACC to monitor changes in the internal calcium level of neurons. A miniscope system was constructed to observe the ACC neuronal activity in free-moving mice. The itching was induced by injecting histamine into the backs of the mice. Simultaneously, in vivo miniscope calcium imaging was used to record mouse behavior with a separate camera (Fig. 1A). Under this setup, the calcium signal of the neurons was clearly observed, and the expression of GCaMP6f in the ACC was confirmed after the experiment (Fig. 1B). As shown in Fig. 1C, the calcium transients of each ACC neuron were observed after histamine injection. First, we investigated whether neurons directly related to itchy sensation were present in the ACC. In rodents, pruriceptive stimuli induce an innate behavioral response to scratching to eliminate factors that cause itchy sensations. Therefore, we assumed that if there were specific neurons directly involved in the itching sensation, changes in calcium transients in this type of neuron would coincide with scratching events, as in the VLO and VTA [5, 6]. However, we did not find any such type of neuron showing calcium transients synchronized with scratching bouts. In addition, this was not observed at the neuronal population level (Fig. 1D). Next, we compared the mean calcium level 1 s before and after the scratching bout. The mean calcium level decreased immediately after scratching bouts (Fig. 1E and Table S1). As mentioned previously, the ACC plays a role in heterologous neurophysiological functions. Thus, we reanalyzed the internal calcium level of each neuron, which was specifically activated only after histamine injection. The results showed differences in calcium levels before and after scratching bouts, which implied that overall neuronal activity was reduced immediately after scratching (Fig. 1F and Table S1).

**Fig. 1:**
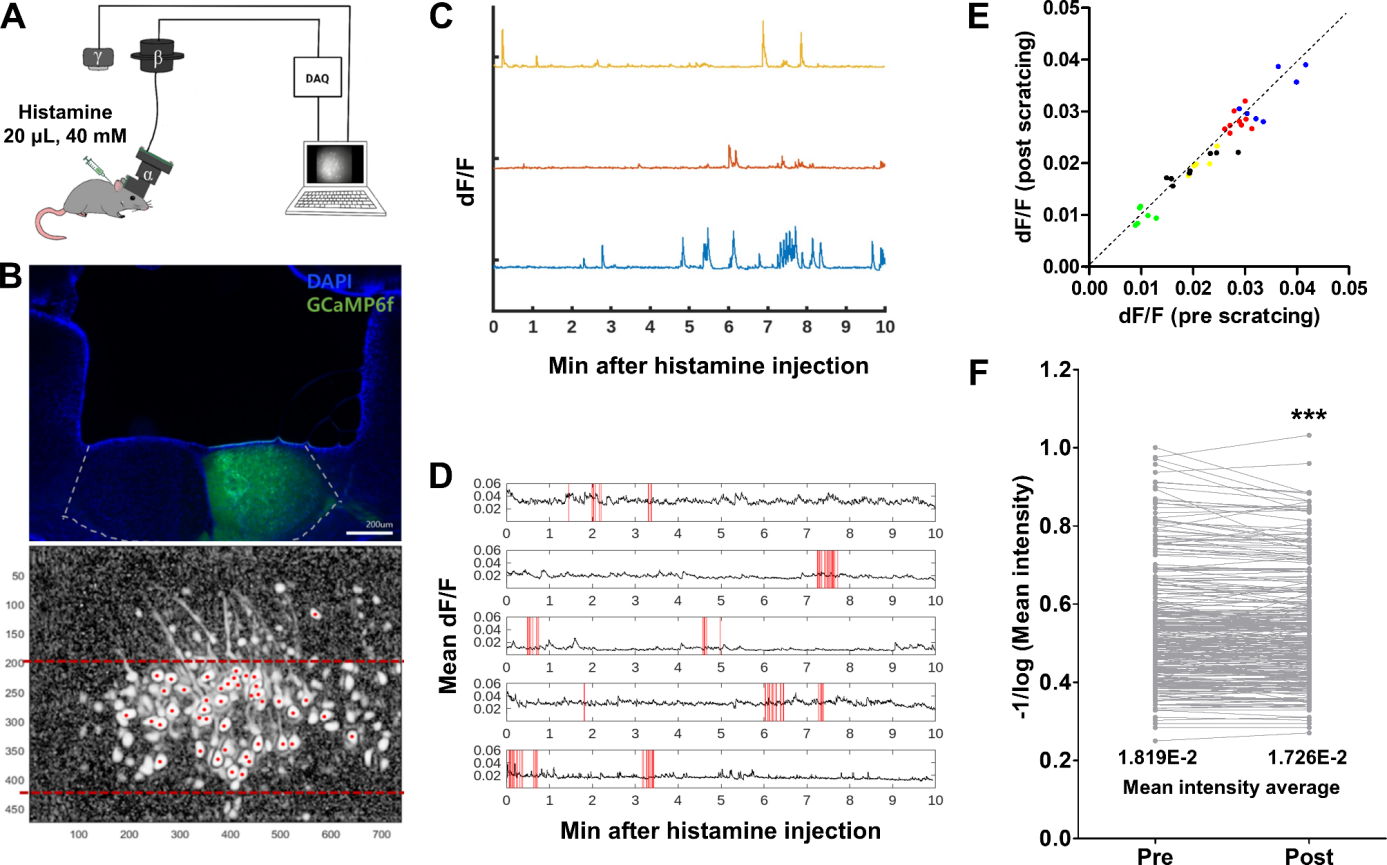
The neuron activity in the ACC is independent of the histamine-induced scratching response. (**A**) The schematic image of the miniscope imaging system. DAQ hardware received the calcium signals from miniscope, and behavior was simultaneously recorded by camera. α: miniscope, β: commutator, preventing the twisting of coaxial cable by rotating, γ: behavior camera. (**B**) The representative image of the ACC after (upper) and during (lower) the miniscope imaging. The region between the two red dash lines is ACC layer 2/3. Some of the dendrites reached toward the ACC layer 5. (**C**) Calcium transients in representative 3 neurons after histamine injection. (**D**) The change in mean dF/F did not depend on the scratching response. Each row represents the mean dF/F of one mouse. Red lines represent time points of scratching bouts. (**E**) Mean calcium level 1 s before and after scratching bouts. Mean intensity was reduced immediate after scratching bouts. Same color represents data come from same animal. (**F**) The calcium level of each neuron was reduced immediate after scratching bouts (n = 287 neurons of 5 mice; D’Agostino & Pearson omnibus normality test (post-pre), p < 0.0001; Wilcoxon signed rank test, *** p < 0.0001)

In this study, we found that a few neurons in the ACC were accurately activated in synchronization with the scratching behavioral response. However, at the population level, the activity of ACC neurons was reduced immediately after the scratching behavioral response. Previous studies found that itch-specific neurons are activated in specific time windows of scratching behavior in the VLO and VTA [5, 6]. These studies were based on the assumption that the scratching response is a behavioral expression of itchiness in mice. Thus, neurons that are specifically activated immediately before the scratching response can possibly mediate the itchy sensation. In this study, we did not find specific neurons showing high activities only immediately before scratching bouts. It implies that ACC may not directly elicit itchiness induced by histamine. However, apart from itchiness, pruritogens can also induce additional internal states, such as anxiety. Previous studies have shown that the ACC enhances anxiety in chronic pain conditions [15–17]. Thus, ACC neurons showing reduced activities immediately after scratching bouts are likely to mediate itch-induced anxiety after histamine injection. Given the hedonic effect of scratching, it is also likely that pleasure immediately after the scratching bout suppresses ACC neurons, thereby reducing itch-induced anxiety [5]. Another possibility is that these ACC neurons may mediate attention to the stimulus. In humans and rodents, the ACC is involved in attention for cognitive and emotional processes [7, 18]. Therefore, the scratching behavior may have distracted attention to the itching stimulus by reducing the activity of these neurons. Further studies are required to fully understand these neurons showing reduced activities immediately after the scratching response. The ACC is the well-known brain region in charge of multimodal function such as sensation, emotion, memory, and cognition. A recently published paper shows that the medial prefrontal cortex neurons involved in remote fear memory recall also mediate pain processing [19]. Functional mapping has not yet been made for individual ACC neurons, but whether the itch-specific neurons of the ACC play a role in other functions, and if they do, it is also important to reveal the mechanism.

## Electronic supplementary material

Below is the link to the electronic supplementary material.

Supplementary Material 1

Supplementary Material 2

## Data Availability

All data generated or analyzed during this study are included in this published article.
