# Supplementary material for: Loosely synchronized activation of anterior cingulate cortical neurons for scratching response during histamine-induced itch
Source: Mol Brain. 2023 Jun 13;16:51. doi: 10.1186/s13041-023-01037-7 (PMC10262506; doi:10.1186/s13041-023-01037-7)
Supplement: Supplementary file 1 — Supplementary Material 1 [file 13041_2023_1037_MOESM1_ESM.docx]

Mar. 30th, 2023

**Loosely synchronized activation of anterior cingulate cortical neurons for scratching response during histamine-induced itch**

Chiwoo Lee^1^, Jihae Oh^1^, Jae-Hyung Lee^2^, Bong-Kiun Kaang^1*^, Hyoung-Gon Ko^3*^

^1^Department of Biological Sciences, College of Natural Sciences, Seoul National University, 1 Gwanak-ro, Gwanak-gu, Seoul 08826, South Korea

^2^ Department of Oral Microbiology, Kyung Hee University, College of Dentistry, Seoul, 02447, South Korea

^3^Department of Anatomy and Neurobiology, School of Dentistry, Kyungpook National University, 2177 Dalgubeol-daero, Daegu 41940, South Korea

*Correspondence: [hgko@knu.ac.kr](mailto:hgko@knu.ac.kr) or kaang@snu.ac.kr

**This file includes:**

**Materials and methods**

**References**

**Materials and Methods**

***Animals***

Male C57BL/6NCrljBgi mice aged between 6–8 weeks were purchased from Samtako Bio Korea. Animals were housed in standard laboratory cages under a 12-h light-dark cycle with access to food and water *ad libitum*. All the experiments were approved by the Institute of Laboratory Animal Resources of Seoul National University (SNU-180409-3).

***Integrated microendoscope imaging***

For *in vivo* 1-photon calcium imaging, integrated microendoscopes called miniscopes were used. A Miniscope V3 was used in these experiments. A CMOS imaging sensor (Aptina, MT9V032) was mounted on a CMOS imaging sensor printed circuit board (PCB) (available at <https://github.com/daharoni/Miniscope_CMOS_Imaging_Sensor_PCB>) and soldered to a coaxial cable (RG-174/U(50Ω). A Luxeon SMD blue LED (P/N LXML-PB01-0030) soldered to an excitation LED PCB was integrated into the main body of the microscope using the CMOS imaging sensor PCB. As optic tools, a 5 mm Dia. x 12.5 mm FL, MgF2 Coated, Achromatic Doublet Lens (Edmund Optics, 49-923), an excitation filter (Chroma, ET470/40×), an emission filter (Chroma, ET525/50m), and a dichroic mirror (Chroma, T495lpxr) were also integrated into the main body of the miniscope. A 3.0‐mm diameter N‐BK7 half-ball lens (Edmund Optics, 47-269) was attached with optical adhesive (Edmond Optics, 55-084) and cured with UV light. The integrated miniscope was linked to a commutator (PANLINK, PSR-C6) to prevent cable coiling.

***Stereotaxic surgeries for calcium imaging***

All the surgeries were performed on C57BL/6N mice. For viral injections, mice (8 weeks) were anesthetized by intraperitoneal injection of a ketamine/xylazine solution and placed in a stereotaxic apparatus (Stoelting Co.). AAV2/1-EF1α-GCaMP6f was injected into the right ACC (AP 0.7 mm, ML 0.25 mm, DV 1.9 mm) using a 33-gauge needle with a Hamilton syringe. After a 2 min rest during which the injection needle’s tip was placed 0.1 mm below the target location, the tip was returned to the target location and 0.5 μL of virus was injected with a 0.125 μL/min flow rate. The needle was slowly removed 7 min after injection. One week after AAV injection, a 2.0-mm diameter GRIN lens (Go!Foton, CLHS200GFT027) was implanted over the ACC. The mice were anesthetized by intraperitoneal injection of ketamine/xylazine and positioned in a stereotactic apparatus. The craniotomy for the GRIN lens was 2.0–2.1 mm in diameter to leave a minimal gap between the GRIN lens and the skull. The cylindrical column of the neocortex was aspirated with saline using a blunt 27-gauge needle until the plane of the subfornical artery was located. After the bleeding stopped, the blood clot was gently removed and a GRIN lens was implanted (DV 1.7 mm) into the hole. Screws were added to the skulls for anchoring. The GRIN lens and screws were fixed using Loctite and dental cement. After curing, a biocompatible silastic elastomer (Kwik-Sil, World Precision Instruments, Berlin, Germany) was added around the GRIN lens to protect it from scratching. Three weeks after GRIN lens implantation, the mice were anesthetized again by intraperitoneal injection of ketamine/xylazine and positioned in a stereotactic apparatus for base plating surgery. A baseplate attached to a miniscope was placed on the GRIN lens and fixed with dental cement at an angle corresponding to the best GCaMP6f signal. The GRIN lens was then covered with a cap with screws on the baseplate until the day of calcium imaging.

***Behavior procedures with miniscopes***

After undergoing all the necessary stereotaxic surgeries, including base plating, the mice were monitored for one week. The mice were individually caged. All the training and testing were conducted during the light cycle. The mice were habituated to the coaxial cable and miniscope for five days without isoflurane anesthesia. Each habituation step lasted for 10 min, with 30 min rest sessions. A separate miniscope was used for each mouse. For the first two days, habituation was hands-only. For the next two days, the mice underwent miniscope attachment habituation, in which the miniscopes were attached to baseplates on their heads with screws, and the mice were delivered to a new cage where they could move freely for 30 s. The miniscopes were then detached and the mice were delivered back to the cage, where they could move freely for another 30 s. This procedure was repeated for 10 min. On the last day of habituation, the mice were anesthetized with isoflurane and the miniscopes were attached. After a 30 min rest, the mice were delivered from a new cage to an anesthesia box for 30 s.

***Calcium imaging of freely moving mice***

On the day of calcium imaging, the mice were habituated to a rack for 30 min with their miniscopes and commutators attached. Calcium imaging was performed during the light cycle, and calcium events were captured using DAQ software (available at https://github.com/daharoni/Miniscope_DAQ_Software) at 15 frames/s, with the CMOS sensor set at maximum gain. To prevent photobleaching, the LED power was set to 3–10%, and each mouse received the same LED power during the calcium imaging sessions. The commutators were connected to a DAQ board such that the CMOS signal could be sent to the software on a computer. After 5 min of baseline imaging, the mice were anesthetized with isoflurane and injected with histamine (40mM, 20μL in saline) into the subcutaneous tissues at the nape of the neck. After a 5 min recovery from isoflurane, the calcium response to histamine was imaged for 10 min. After calcium imaging, mice were detached from their miniscopes and returned to their home racks. The LED was turned off except during the imaging steps.

***Calcium imaging data acquisition, processing, and cell sorting***

Calcium transients captured by the DAQ software and hardware (Labmaker or Sierra Circuits, v3.2) were saved in the .avi video format. These videos were processed using MIN1PIPE (1), and individual session videos were combined into a movie. The combined video was subjected to several analyses, including preprocessing and neural activity identification. CellReg was used to track the detected neuronal population before and after histamine injection (2). Neural population classification and signal processing were performed using custom MATLAB code.

**References**

1. Lu J, Li C, Singh-Alvarado J, Zhou ZC, Fröhlich F, Mooney R, et al. MIN1PIPE: A Miniscope 1-Photon-Based Calcium Imaging Signal Extraction Pipeline. Cell Rep. 2018 Jun 19;23(12):3673–84.

2. Sheintuch L, Rubin A, Brande-Eilat N, Geva N, Sadeh N, Pinchasof O, et al. Tracking the Same Neurons across Multiple Days in Ca2+ Imaging Data. Cell Rep. 2017 Oct 24;21(4):1102–15.
